# Targeting P2X Receptors—Current Progress in Sepsis

**DOI:** 10.1155/ijin/1083543

**Published:** 2025-09-24

**Authors:** Lan Luo, Qian Zhao, Yunfen Tian, Meisha Sun, Mazhong Zhang, Bin Wang

**Affiliations:** ^1^Department of Anesthesiology, Guizhou Provincial People's Hospital, Guiyang, China; ^2^The First School of Clinical Medicine, Zunyi Medical University, Zunyi, China; ^3^Department of Anesthesiology, Shanghai Children's Hospital, Shanghai Jiao Tong University School of Medicine, Shanghai, China

**Keywords:** multiple organ dysfunction, P2X receptors, purinergic receptor, sepsis

## Abstract

Sepsis is defined as life-threatening organ dysfunction caused by a dysregulated host response to infection. Inflammation, as the main pathophysiological mechanism, runs through the whole course of sepsis. Notably, P2X receptors have the capacity to mediate inflammation, nerve signaling, and thrombosis, which underscores their pivotal role in the progression of sepsis. The goal of this study is to review the specific role of the P2X family in the pathogenesis of sepsis in various organs in light of currently available evidence.

## 1. Background

Sepsis is a life-threatening multiorgan dysfunction caused by immune dysregulation in response to infection [1]. Sepsis, a leading cause of mortality in intensive care units, poses a significant global health burden. In 2017, an estimated 48.9 million sepsis cases and 11.0 million sepsis-related deaths were reported worldwide, accounting for 19.7% of global mortality [2]. Currently, the core pathological mechanism believed to underlie sepsis is immune dysfunction. The management of sepsis mainly centers on supportive and symptomatic treatments. This encompasses antibiotic therapy to combat infections, measures to stabilize hemodynamics, and strategies for preserving organ function [3]. Effective targeted therapies are still being explored.

The P2X receptor is a nonselective ion channel with broad distribution across various cell types, including neurons, bone cells, exocrine glands, endothelial cells, and muscle tissues [4, 5]. P2X plays significant roles in diverse biological processes, including tumor progression, immune regulation, hemolysis, and neurodevelopment [6]. Due to their widespread distribution and low ion selectivity, P2X are implicated in multiple aspects of sepsis pathogenesis, particularly in modulating immune system activation and organ-specific responses. Although numerous studies have explored P2X agonists, antagonists, and animal models, current research remains largely confined to initial discovery and validation phases. This underscores the need for more in-depth mechanistic investigations to fully elucidate the roles of P2X in the pathogenesis of sepsis [7]. Knockout of P2X significantly impacts the survival rate of mice with severe infections [8]. Moreover, P2X has been demonstrated to play a role in influencing the prognosis of sepsis linked to neonatal meningitis-associated *Escherichia coli* (NMEC) [9]. Indeed, numerous studies have investigated the relationship between P2X and sepsis from diverse perspectives. However, these studies are often fragmented and inconsistent. This study aims to systematically summarize the research literature pertaining to the role of P2X in the pathogenesis of sepsis by organizing findings within the context of individual organ systems.

## 2. P2 Purinergic Receptors

Purinergic receptors are categorized into P1 and P2 receptors. P1 receptors are activated by adenosine, while P2 receptors respond to ATP and its metabolites, ADP and AMP. P2 receptors are further classified into two main subtypes: P2Y receptors, which are G protein-coupled receptors, and P2X receptors, which function as ligand-gated nonselective ion channels. To date, eight P2Y subtypes and seven P2X subtypes have been identified [6]. This study mainly focuses on the P2X subtypes.

The P2X1 receptor primarily functions in the urinary, immune, and cardiovascular systems [10]. It is highly expressed in smooth muscle, platelets, and neutrophils, where it plays a key role in mediating thrombus formation and inflammatory responses [11]. The P2X2 receptor is predominantly expressed in neuronal cells, particularly in taste buds and the cochlea [12]. The P2X2 receptor, in conjunction with the P2X3 receptor, facilitates the transmission of neural electrical signals, playing a critical role in nociceptive signaling and the sensory regulation of visceral functions [13]. The P2X4 receptor is expressed in the nervous and cardiovascular systems and is closely linked to the pathogenesis of chronic neuropathic pain [14]. The P2X5 receptor may contribute to modulating inflammatory responses [15]. The P2X6 receptor has been scarcely studied, with limited research available. In contrast, the P2X7 receptor is widely distributed across multiple organs, including the liver, intestines, blood vessels, and bone [16]. Among all P2 receptors, P2X7 is most strongly associated with the regulation of inflammatory responses [17]. In addition, P2X7 is also closely related to disorders of coagulation [18] and diseases of the nervous system [19].  Table 1 summarizes the organ-specific distribution of each P2X receptor in humans.

A balanced immunological status helps regulate inflammatory responses, whereas a central pathological hallmark of sepsis is immune dysregulation, typically characterized by early hyperactivation followed by late-stage immune paralysis, both of which contribute to multiple organ dysfunction [21]. Key features of sepsis-related immunosuppression include elevated levels of anti-inflammatory cytokines, exhaustion, and apoptosis of CD4^+^ and CD8^+^ T cells, B cells, natural killer cells (NK cells), and dendritic cells. Additionally, there is an upregulation of PD-1 and a downregulation of HLA-DR. Given these complex immunological alterations, precision immunotherapy targeting specific immune pathways represents a promising therapeutic strategy for sepsis management [22]. Several antagonists targeting P2X receptors have been developed, demonstrating promising results in animal models.  Table 2 lists some common P2X antagonists.

## 3. Liver Injury

The liver is a vital organ in metabolism and immune regulation. Patients with concurrent sepsis and cirrhosis exhibit a fourfold increase in mortality compared to those without these conditions [32]. Sepsis-induced liver injury primarily results from hemodynamic instability, with inflammatory hepatocyte dysfunction also playing a significant role [33].

**Figure 1:**
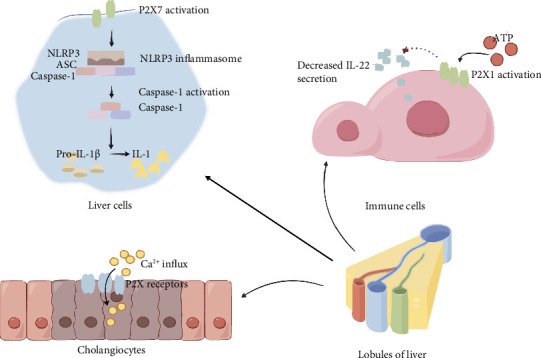
Overview of liver injury in sepsis. The liver can be divided into hepatic lobules. P2X1 significantly inhibits the secretion of growth-promoting factor interleukin-22 (IL-22) in vitro in immune cells; P2X7 mainly promotes inflammation through the P2X7-NLRP3-Caspase-1 pathway; P2X on the apical membrane is activated by ATP and mediates intracellular Ca^2+^ influx, affecting cell metabolism and proliferation.

All P2X receptor subtypes are expressed in the liver, with P2X4 and P2X7 being the most predominantly expressed [34]. Under physiological conditions, P2X receptors play a role in essential liver functions, including bile formation [35, 36].  Figure 1 provides an overview of the important mechanisms of P2X in liver injury.

P2X1 plays dual roles in the pathogenesis of sepsis. On one hand, P2X1 exerts a protective effect in endotoxemia by inhibiting systemic neutrophil activation, thereby mitigating oxidative stress, coagulation, and subsequent organ injury [37]. P2X1 downregulates stimulator of interferon genes (STING) and NK cells [38, 39]. STING is a signaling molecule activated by cyclic GMP–AMP synthase (cGAS). Upon activation, STING drives the production of type I interferons and can induce cell death [40]. On the other hand, P2X1 significantly suppresses the secretion of the growth-promoting factor interleukin-22 (IL-22) in vitro, thereby impairing the regeneration and repair of damaged hepatocytes during the late stages of sepsis [41].

P2X4 is implicated in fibrosis and regeneration following liver injury. The liver is organized into hepatic lobules, which consist of various cellular components, including sinusoidal endothelial cells, Kupffer cells, stellate cells, hepatic myofibroblasts (hMF), and bile duct epithelial cells. Notably, stellate cells and myofibroblasts can undergo mutual transformation. P2X4 is predominantly expressed on stellate cells and cholangiocytes, playing a critical role in these processes [42]. ATP is released from cholangiocytes into bile, where it activates P2X4 receptors located on the apical membrane, triggering intracellular Ca^2+^ influx [36]. Simultaneously, Ca^2+^ serves as a key cation for the activation of phosphatidylinositol 3-kinase (PI3K) and lysosomal exocytosis in hMF [43]. This signaling receptor plays a pivotal role in regulating cellular proliferation [44]. Thus, P2X4 serves as a critical link between bile metabolism and the development of liver fibrosis [45].

P2X7 primarily drives inflammation through two key pathways:

1. P2X7-NLRP3-Caspase-1 pathway: This is the most significant signaling pathway through which P2X7 promotes inflammation. Indeed, beyond sepsis, P2X7 plays a crucial role in various inflammatory diseases (such as Crohn's disease, chronic hepatitis, etc.) and chronic liver fibrosis by activating this pathway [46]. NLRP3 is a critical sensor in the innate immune system, while caspase-1, a cysteine protease, plays a key role in converting pro-interleukin-1β (pro-IL-1β) and pro-interleukin-18 (pro-IL-18) into their active forms [47, 48]. When cells undergo death, a significant amount of ATP is released into the extracellular space. P2X7 detects these changes in ATP concentration and becomes activated, subsequently triggering NLRP3. This cascade ultimately leads to caspase-1-dependent release of IL-1β and IL-18, initiating an inflammatory response [49, 50]. Modulating this signaling pathway using electroacupuncture may alleviate liver inflammation and, through the gut–brain axis, potentially ameliorate symptoms of depression [51]. It is noteworthy that since NLRP3 activation is dependent on P2X7, the regulation of P2X7 on macrophages helps prevent excessive inflammatory responses [52]. Thus, while P2X7 promotes inflammatory responses, it also plays a crucial role in limiting excessive immune activation and maintaining immune homeostasis.
2. P2X7 interacts with Pannexin 1 (Panx1), a nonselective membrane channel that facilitates the passage of ATP and other macromolecules, such as K^+^, Cl^−^, Ca^2+^, and glutamate [53]. Panx1 can be activated by caspase-11, an inflammation-related enzyme that is typically not expressed under normal conditions but is induced by lipopolysaccharide (LPS) during sepsis [54]. When caspase-11 activates Panx1, it triggers cell lysis, leading to the release of large amounts of ATP [55]. This released ATP further activates P2X7, initiating a cascade of subsequent inflammatory responses. Notably, the anti-inflammatory properties of the renowned Chinese herbal medicine licorice are attributed to its main active component, the glycyrrhizin acid derivative carbenoxolone, which directly inhibits the P2X7/Panx-1 pathway [56]. However, this process does not solely signify an absolute proinflammatory response, as the release of large amounts of ATP into the cytoplasm can counteract hyperosmotic stress in the extracellular environment [57]. In the early stages of sepsis, the interaction between P2X7 and Panx1 can positively regulate interleukin-33 (IL-33) [58]. When tissue damage occurs, IL-33 acts as an alarm signal by binding to suppression of tumorigenicity 2 receptors (ST2 receptors), stimulating mast cells, T cells, and other immune cells to produce inflammatory factors [59]. The P2X7-Panx1-IL-33 axis can modulate the population of T cells expressing ST2 receptors in the liver, aiding sepsis patients in more rapidly overcoming the phase of heightened inflammatory stress [60].

## 4. Sepsis-Associated Encephalopathy (SAE)

**Figure 2:**
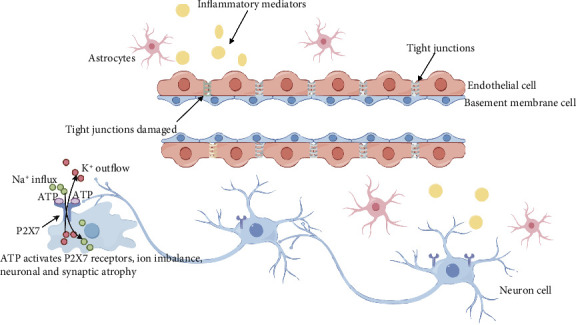
Overview of sepsis-associated encephalopathy. Astrocytes release inflammatory mediators in response to systemic inflammation, leading to neuronal and endothelial cell damage. This process results in the release of large amounts of ATP, activating P2X receptors and causing ion flow disruption. Consequently, tight junctions between cells are compromised, and the blood–brain barrier becomes dysfunctional.

SAE is a diffuse brain dysfunction secondary to sepsis. A significant number of patients with sepsis develop neurological complications in later stages [61]. The primary pathogenesis of SAE involves inflammation, oxidative stress damage, and disruption of the blood–brain barrier [62]. P2X and septic encephalopathy are closely related, as shown in Figure 2.

### 4.1. Blood–Brain Barrier (BBB) Disruption

BBB is formed by microvascular endothelial cells, with tight junctions (TJs) serving as the critical structural components of its junctional complex [63, 64]. In sepsis, the inflammatory response mediated by P2X activation leads to significant atrophy of endothelial cells and disruption of TJs [65]. P2X7 can mediate leukocyte adhesion and microglial trafficking, thereby linking neurovascular inflammation to brain injury [66]. The recreational drug 3,4-methylenedioxymethamphetamine (MDMA; “ecstasy”) [67] and Maf1 [68] can affect BBB permeability by antagonizing P2X7.

### 4.2. Microglial Activation

Microglia are the most important immune cells in the nervous system [69]. In vitro experiments have demonstrated that inhibiting P2X7 can reduce the production of IL-1β, TNF-α, and interleukin-10 (IL-10), while also reversing damage caused by microglial activation in response to inflammatory stimuli in brain tissue [70, 71]. During SAE, proinflammatory signals are transmitted across the BBB and subsequently activate microglia. These activated microglia release inflammatory mediators that stimulate neural cells to generate excessive amounts of ATP. The elevated ATP levels further potentiate the activation of P2X7 receptors, creating a positive feedback loop in the neuroinflammatory response [72]. The activated P2X7 receptor facilitates the release of interleukin-6 (IL-6), a cytokine predominantly associated with chronic inflammatory processes. This released IL-6 subsequently initiates the activation of signal transducer and activator of transcription 3 (STAT-3), thereby triggering downstream signaling cascades [73]. STAT-3 interacts directly with the transcription factor nuclear factor-κB (NF-κB) to synergistically regulate the production of diverse inflammatory cytokines. This molecular interplay establishes the P2X7/IL-6/STAT-3 signaling axis as a critical pathway in the pathogenesis of SAE, suggesting its potential as a therapeutic target for SAE [74].

### 4.3. Neuronal Cell Dysfunction

P2X7 is a microporous channel protein permitting the passage of large ions like Na^+^, K^+^, and Ca^2+^ [75]. During SAE, substantial activation of P2X7 leads to an imbalance of ions across the cell membrane of neuronal cells. This ion gradient not only directly impairs cell osmolarity but also damages mitochondria. Subsequently, the damaged inner mitochondria release numerous apoptosis-related proteins. These proteins then enter the cytoplasm and trigger the activation of apoptosis signaling pathways [76]. Inhibiting P2 receptors curbs the production of reactive oxygen species (ROS) in the hippocampus and cortex and reverses cognitive impairment [70].

In addition to its established roles in immune activation, the P2X7 receptor's characteristic pore formation may contribute to neuronal cell dysfunction. While the exact mechanisms remain under investigation, this pore-mediated effect could potentially exacerbate neuroinflammation and cognitive impairment in septic patients [77]. Further studies are needed to fully elucidate this pathway's clinical significance.

## 5. Intestinal Injury

### 5.1. Intestinal Barrier

Compared with other organs, the intestine has a special barrier structure—the intestinal barrier. The intestinal barrier is a semi-permeable membrane mainly composed of four parts: the microbial barrier, the chemical barrier, the physical barrier, and the immune barrier [78]. Among them, various immune cells make up the immune barrier [79], especially T cells [80]. During the activation of P2X7 on T cells, two independent pathways directly induce T cell death:

1. Phosphorylation of extracellular signal-regulated kinase (ERK1/2). ERK1/2 is a key signaling node in the mitogen-activated protein kinases (MAPK) pathway cascade and is associated with cell differentiation and proliferation [81].
2. T cell contraction. P2X7 is highly expressed in most intestinal αβ and γδ T cells, including Th1 and Th17 cells [78]. P2X7 can trigger nicotinamide adenine dinucleotide NAD^+^-dependent ADP ribosylation. This NAD^+^-dependent ADP ribosylation is a signal transduction system that is ubiquitously present across diverse biological entities and exerts functions in aspects such as virulence manifestation and immune responses [82]. For example, retinoic acid (RA) activates the RA-responsive enhancer region in the P2X7 gene, which in turn induces the expression of P2X7 in Th1 and Th17 cells. This induction leads to the contraction of Th1 and Th17 cells, ultimately contributing to the maintenance of intestinal immune homeostasis [83].

### 5.2. Intestinal Hemorrhage

Platelets and P2X1 are necessary to maintain intestinal vascular integrity [84, 85]. Studies have demonstrated that the plasma levels of granulocyte colony-stimulating factor (G-CSF) are elevated in P2X1-deficient mice. G-CSF serves as a crucial factor in hematopoietic development and is associated with pathological hematopoietic differentiation [86]. Excessive elevation of G-CSF levels may lead to the formation of atypical neutrophils and fibrin-rich thrombi, resulting in chronic hemorrhage during the initial phase and progressing to macrocytic anemia in the advanced stage [87]. In cases of sepsis complicated by congestive heart failure, P2X1 receptor-mediated intestinal capillary constriction occurs as a compensatory mechanism to maintain perfusion of vital organs, which consequently elevates the risk of intestinal hemorrhage [88]. P2X4 receptors demonstrate predominant expression within the crypts of the colon, with particularly high density observed in the epithelial lining [89]. P2X4 receptors are functionally associated with calcium ion influx and glycolytic regulation. The enzymatic activity of ectonucleoside triphosphate diphosphohydrolase (E-NTPD), which catalyzes ATP hydrolysis, exerts inhibitory effects on P2X4 receptor signaling, thereby mediating both anti-inflammatory and hemostatic responses [90].

### 5.3. Peristaltic Dysfunction

Purinergic signaling pathways do not participate in mechanosensory transduction under normal physiological conditions in the small intestine, but they play a crucial role in mediating mechano-hypersensitivity following infectious episodes [91]. P2X2 is expressed in particular subtypes of enteric neurons, such as inhibitory motor neurons, noncholinergic motor neurons, and intrinsic primary afferent neurons. These neurons play a role in generating rapid excitatory postsynaptic potentials in intestinal neurons [92, 93]. In sepsis, P2X2 is activated by a substantial quantity of ATP, and this activation further induces a movement disorder in intestinal glial cells [94]. Experiments have demonstrated that in rat models of intestinal ischemia-reperfusion injury, the density of P2X2 in neurons of the ileal plexus and submucosal plexus is significantly decreased. This decrease led to alterations in intestinal motility [95, 96]. P2X3 is expressed by the sensory fibers located beneath the intestinal epithelium. When the intestine undergoes dilation, P2X3 relays signals to the pain center within the central nervous system via the intermediate neurons in the spinal cord. Additionally, it takes part in intestinal peristalsis. Notably, there is a distinct positive correlation between its expression level and the activity of sensory nerves [97–99].

## 6. Kidney Injury

The kidney is one of the most commonly affected organs in sepsis, and patients with renal dysfunction are predicted to have increased mortality [100]. Similar to liver damage, sepsis-induced kidney injury is mostly caused by hemodynamic changes and inflammation [101, 102]. It is noteworthy that the kidney possesses characteristic physiological defense mechanisms, namely autoregulation and glomerular feedback. Under physiological conditions, a direct correlation exists between the autoregulation of renal perfusion pressure, renal vascular resistance, and the concentration of ATP in the renal interstitial fluid [103, 104]. Studies have demonstrated that purinergic P2 antagonists are capable of alleviating angiotensin II-dependent hypertension. Overall, P2X receptors play a role in regulating renal vasoconstriction, tubular function, inflammation, and renal fibrosis [105, 106].

Based on autoradiography findings, P2X1 is predominantly situated in the vascular smooth muscle of the internal renal arteries, such as the arcuate artery, interlobular artery, and afferent arteriole. It plays a crucial role in the autoregulation of the afferent arteries [107, 108]. Using immunosuppressants to keep P2X1 in an inactive state may contribute to the maintenance of renal autoregulation [109].

P2X4 is expressed in large quantities in all segments of the nephron, but it mainly works in the distal segment of the nephron [110]. The activation of P2X4 aids in regulating the activity of the epithelial sodium channel (ENac). When the filtrate osmolarity is low, P2X4 increases the activity of ENac; conversely, when the filtrate osmolarity is high, P2X4 downregulates its activity. In this way, P2X4 enables the real-time and precise regulation of blood pressure [111, 112].

P2X6 is present in the distal convoluted tubule (DCT). The DCT is responsible for the reabsorption of Mg^2+^ and Na^+^. However, experimental evidence indicates that P2X6 does not exert a significant impact on electrolyte balance [113].

P2X7 has a relatively low distribution in glomeruli and is mainly located in the renal tubules and renal interstitium. It is capable of promoting kidney inflammation via the classic P2X7/NLRP3/caspase-1 pathway. The newly discovered P2X7 antagonist 14a can inhibit the activation of the NLRP3 inflammasome, thus impeding kidney damage in septic mice [114, 115]. Expression of cleaved caspase-1, IL-1, and IL-18 is expected to be an effective treatment [30]. Furthermore, epithelial-calcium mucin is a cell adhesion molecule that mediates adhesion between epithelial cells. It can be downregulated by P2X7, which leads to renal tubular damage [116]. However, the effects of P2X7 are still controversial. Studies have shown that direct knockout of P2X7 did not lead to a reduction in proteinuria, renal tubular damage, renal macrophage accumulation, and renal perivascular fibrosis, suggesting that P2X7 may have little impact on renal vascular damage [117].

## 7. Lung Injury

**Figure 3:**
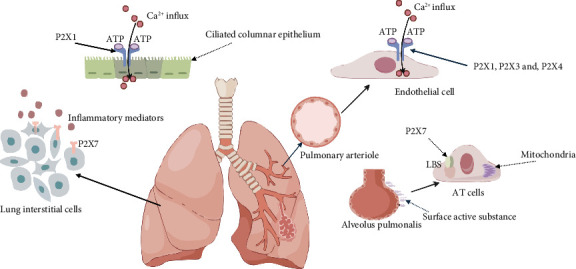
Overview of lung injury in sepsis. The opening of P2X1 channels increases Ca^2+^ entry into bronchial mucosal epithelial cells causing bronchial contraction. P2X1, P2X3 and P2X4 on the surface of vascular endothelial cells were associated with pulmonary artery vasoconstriction. P2X7 is located on the surface of lamellar bodies (LBs) in alveolar epithelial cells (AT cells) and assists in the secretion of surface-active substances. The ion concentration gradient caused by P2X caused mitochondrial damage. Activation of P2X7 receptors on pulmonary interstitial cell membranes upregulates the secretion of inflammatory cytokines through the classic pathways.

Lung injury occurs in nearly 50% of patients with sepsis [118]. The principal pathologic mechanisms involve acute inflammation and damage to the endothelial barrier, as well as pulmonary edema and pulmonary hypertension resulting from alveolar epithelial injury [118, 119]. On the surface of alveolar macrophages, there are diverse P2X receptors. Once activated by ATP, these receptors trigger the release of a vast amount of cytokines, which in turn results in lung damage [120].  Figure 3 summarizes the mechanisms of P2X-related lung injury in sepsis.

Purine energy signaling serves as a crucial determinant in the regulation of pulmonary vascular physiology. P2X1 facilitates the influx of extracellular Ca^2+^, thereby causing the constriction of pulmonary blood vessels and tracheal smooth muscle [121, 122]. CD39, an extracellular nucleotide hydrolase, functions as an antagonist of P2X1. By counteracting the vasoconstrictive effects of P2X1, it effectively alleviates pulmonary hypertension [123]. Nevertheless, a prolonged elevation of CD39 may heighten the responsiveness to P2X, thereby exacerbating lung inflammation further [124].

The expression of P2X3 and P2X2 in the sensory fibers of the nerves within the lungs leads to bronchial constriction and the local release of proinflammatory neuropeptides. These processes are closely associated with chronic inflammation and coughing [125–127]. So the administration of the P2X2/3 antagonist DT-0111 may relieve chronic cough [128, 129]. Transient receptor potential cation channel, subfamily V, member 4 (TRPV4), a nociceptor, is capable of interacting with ATP-P2X3 to engage in the osmotic pathway of airway sensory nerve reflexes. This makes TRPV4 a novel and promising therapeutic target for addressing the high reactivity of airway neurons and cough, potentially opening new avenues for treating related respiratory conditions [130]. Mitochondrial dysfunction, ROS, and protein kinase C translocation and activation are stimulators of this signaling pathway [131, 132].

P2X4 is expressed on the membrane of lamellar bodies (LBs) in alveolar epithelial cells (AT cells). LBs are large lysosome-derived secretory organelles. They are associated with the secretion of surfactant substances and play a role in maintaining alveolar fluid balance [133]. When there is an excess of pulmonary interstitial ATP, the P2X4 receptors on the surface of type II alveolar epithelial (AT II) cells become desensitized. This desensitization leads to a decrease in surfactant production. As a result, the alveoli collapse, and an imbalance occurs in the exchange of alveolar fluid [134, 135].

The activation of P2X7 is essential for inflammasome activation. As previously stated, P2X7 predominantly exerts its proinflammatory effect via the classical NLRP3 inflammasome and Panx1. Once P2X7 is knocked out, lung inflammation is markedly diminished. For example, the P2X7 antagonist A-438079 mitigates oxidative stress in the lungs by maintaining a balance between tissue oxidants and antioxidants [29, 136].

## 8. Limitations of Experimental Sepsis Models

In light of the MQTiPSS (Minimum Quality Threshold in Pre-Clinical Sepsis Studies) guidelines [137], while the LPS-induced model has been widely used in sepsis research due to its simplicity and reproducibility, it is important to acknowledge its limitations in replicating the complexity of human sepsis. The LPS model primarily mimics the hyperinflammatory phase of sepsis via a single Toll-like receptor 4 (TLR4)-dependent pathway. This reductionist approach fails to account for critical aspects of human sepsis, such as pathogen heterogeneity, immunosuppressive phases, and metabolic dysregulation. Consequently, the MQTiPSS guidelines explicitly recommend against relying solely on endotoxemia models and advocate for the use of polymicrobial infection models (e.g., CLP) that better approximate the clinical scenario.

Among the studies cited in this manuscript, references [34, 53, 57, 66] employed the LPS-induced endotoxemia model, whereas references [68, 69, 71] utilized the cecal ligation and puncture (CLP) model. These methodological differences likely explain key inconsistencies in the literature. For instance, regarding survival outcomes, P2X7 knockout decreased survival in an α-hemolysin-producing *E. coli* model [8], whereas P2X7 antagonism improved survival in LPS-induced sepsis [27]. For organ injury, P2X7 inhibition protected against renal dysfunction in both CLP [28] and LPS models [30], demonstrating dose-dependent efficacy in septic AKI or lung injury [29] with good safety profiles.

This methodologic assessment raises two important reflections: (1) employing multiple sepsis models to validate findings and (2) reporting detailed methodological parameters (e.g., LPS dose/route, CLP needle gauge, fluid resuscitation protocols).

## 9. Conclusions

P2X is a low-selective ligand-gated ion channel receptor, enabling the passage of large ions like Na^+^, K^+^, and Ca^2+^. The low selectivity of the gated ions determines the multiplicity of its physiological effects. P2X plays a pivotal role in blood clotting, inflammation, and nerve conduction. Sepsis, which mostly occurs secondary to severe systemic infections, often leads to multiple organ dysfunction. In the early stages of sepsis, P2X can function as nociceptors and initiate an immune response, serving as a “first stop” for immune defense. Nevertheless, the proinflammatory response triggered by P2X itself may result in excessive immune activation and subsequent tissue damage. In the late stages of sepsis, P2X also sustains the activity of the body's immune system. Currently, numerous animal experiments have demonstrated that the effects of P2X are complex and diverse, varying with the disease course and the affected organs in patients with sepsis. Targeting P2X holds great potential as an effective treatment approach for sepsis. Currently, clinical trials of P2X receptor antagonists are still in the exploratory stage, with promising potential to achieve positive results and be applied in clinical practice as soon as possible. For example, JNJ-55308942, these studies primarily focus on the clinical efficacy, safety, and pharmacokinetics of the drugs. (https://clinicaltrials.gov/study/NCT05328297; NCT03437590; NCT03151486; NCT03547024). On the whole, P2X may be a promising target for treatment of sepsis.

## Figures and Tables

**Table 1:** Tissue distribution and cellular localization of P2X.

Receptor	Location
P2X1	Smooth muscle: heart, bladder, vas deferens, and arteries (vascular smooth muscle) Blood cells: platelets, mast cells, and lymphocytes Nervous system: astrocytes and spinal cord
P2X2	Nervous system: retina, carotid body, brain, enteric nervous system, cochlea, autonomic neurons, and sensory neurons
P2X3	Sensory nervous system, trigeminal ganglia, pelvic nerve, dorsal root ganglia, and taste buds
P2X4	Sensory nervous system, olfactory bulb, hypothalamus, cerebellum, GABAergic neurons of the striatum and substantia nigra, and retinal ganglia
P2X5	Immune system: T cells and B cells
P2X6	B cells and heart
P2X7	Ubiquitous: brain (cerebral cortex, piriform cortex, ependymal cells, microglia, lateral septal nucleus, oligodendrocytes, hippocampal pyramidal cells, and other neurons), retina, olfactory nucleus, salivary glands (parotid and submandibular glands), lacrimal glands, Schwann cells, blood cells (erythrocytes, monocytes, macrophages, granulocytes, mast cells, and B and T lymphocytes), thymus, tonsils, bone marrow, bone (osteoblasts and osteoclasts), fibroblasts, dendritic cells, keratinocytes, lung, prostate, testis, heart, liver, skeletal muscle, pancreas, and kidney

*Note:* Reference [20].

**Table 2:** Key purinergic receptor antagonists: mechanisms of action, experimental/therapeutic applications, and clinical trial status.

Receptors	Antagonists	Mechanism of action and potential use	Status
P2X1	Salicylamide derivatives, including PSB-2014, NF023 and NF479, MRS2159	A kind of small, uncharged molecules, which act as negative allosteric modulators [7]	Preclinical
P2X2	PPADS, Reactive Blue 2, TNP-ATP, and suramin	A kind of nonselective P2X2 receptor antagonists [7]	Preclinical
P2X3	Eliapixant (BAY1817080), BLU-5937 Gefapixant	Treats chronic cough [23] Ameliorates postinfarct cardiac dysfunction and autonomic nervous imbalance [24]	Clinical Clinical
P2X4	NP-1815-PX 5-BDBD	Inhibition of bronchial constriction [25] Suppresses microglial activation and subsequent cytokine expression after brain injury [26]	Preclinical Preclinical
P2X5	None		
P2X6	None		
P2X7	A-438079 14a A740003 AZD9056 JNJ-55308942, JNJ-54175446 [7]	Inhibits circulating RNA (circ_0001679, circ_0001212) and mRNA (PLN, CDH2, and NPRL3) [27], ameliorates renal dysfunction [28], and alleviates oxidative stress of the lung [29] Suppresses NLRP3 inflammasome activation [30] Inhibits ERK/NF-κB pathways [31] treat rheumatoid arthritis [7] Treats depression and bipolar disorder [NCT05328297]; interaction with cytochrome P450 [NCT03547024]	Preclinical Preclinical Preclinical Clinical Clinical

## Data Availability

Data sharing is not applicable to this study, as no new data were created or analyzed in this study.

## Nomenclature

MDMA; “ecstasy”: 3, 4-Methylenedioxymethamphetamine
AT cell: Alveolar epithelial cell
BBB: Blood–brain barrier
CLP: Cecal ligation and puncture
cGAS: Cyclic GMP–AMP synthase
DCT: Distal convoluted tubule
E-NTPD: Ectonucleoside triphosphate diphosphohydrolase
ENac: Epithelial sodium channel
ERK1/2: Extracellular signal-regulated kinase
G-CSF: Granulocyte-colony stimulating factor
hMF: Hepatic myofibroblasts
LBs: Lamellar bodies
LPS: Lipopolysaccharide
IL-6: Interleukin-6
IL-10: Interleukin-10
IL-22: Interleukin-22
IL-33: Interleukin-33
MQTiPSS: Minimum Quality Threshold in Pre-Clinical Sepsis Studies
MAPK: Mitogen-activated protein kinase
NK cells: Natural killer cells
NMEC: Neonatal meningitis-associated *Escherichia coli*
NAD^+^: Nicotinamide adenine dinucleotide
P2X: P2X receptor
P2X1-7: P2X1-7 receptor
P2Y: P2Y receptor
Panx1: Pannexin 1
PI3K: Phosphatidylinositol 3-kinase
Pro-IL-1β: Pro-interleukin-1β
Pro-IL-18: Pro-interleukin-18
ROS: Reactive oxygen species
RA: Retinoic acid
SAE: Sepsis-associated encephalopathy
STING: Stimulator of interferon genes
STAT-3: Signal transducer and activator of transcription-3
ST2 receptors: Suppression of tumorigenicity 2 receptors
TJs: Tight junctions
NF-κB: Nuclear factor-κB
TRPV4: Transient receptor potential cation channel, subfamily V, member 4

## References

[1] Singer M., Deutschman C. S., Seymour C. W. (2016). The Third International Consensus Definitions for Sepsis and Septic Shock (Sepsis-3). *JAMA*.

[2] Rudd K. E., Johnson S. C., Agesa K. M. (2020). Global, Regional, and National Sepsis Incidence and Mortality, 1990-2017: Analysis for the Global Burden of Disease Study. *The Lancet*.

[3] Evans L., Rhodes A., Alhazzani W. (2021). Surviving Sepsis Campaign: International Guidelines for Management of Sepsis and Septic Shock 2021. *Intensive Care Medicine*.

[4] Liu J. P., Liu S. C., Hu S. Q. (2023). ATP Ion Channel P2X Purinergic Receptors in Inflammation Response. *Biomedicine & Pharmacotherapy*.

[5] Khakh B. S., Alan North R. (2006). P2X Receptors as Cell-Surface ATP Sensors in Health and Disease. *Nature*.

[6] Burnstock G. (2006). Pathophysiology and Therapeutic Potential of Purinergic Signaling. *Pharmacological Reviews*.

[7] Illes P., Müller C. E., Jacobson K. A. (2021). Update of P2X Receptor Properties and Their Pharmacology: IUPHAR Review 30. *British Journal of Pharmacology*.

[8] Greve A. S., Skals M., Fagerberg S. K. (2017). P2X(1), P2X(4), and P2X(7) Receptor Knock Out Mice Expose Differential Outcome of Sepsis Induced by α-Haemolysin Producing *Escherichia coli*. *Frontiers in Cellular and Infection Microbiology*.

[9] Chambers C. A., Dadelahi A. S., Moley C. R., Olson R. M., Logue C. M., Skyberg J. A. (2022). Nucleotide Receptors Mediate Protection Against Neonatal Sepsis and Meningitis Caused by Alpha‐Hemolysin Expressing *Escherichia coli* K1. *The FASEB Journal: Official Publication of the Federation of American Societies for Experimental Biology*.

[10] Bennetts F. M., Mobbs J. I., Ventura S., Thal D. M. (2022). The P2X1 Receptor as a Therapeutic Target. *Purinergic Signalling*.

[11] Burnstock G., Knight G. E. (2004). Cellular Distribution and Functions of P2 Receptor Subtypes in Different Systems. *International Review of Cytology*.

[12] Finger T. E., Danilova V., Barrows J. (2005). ATP Signaling is Crucial for Communication From Taste Buds to Gustatory Nerves. *Science*.

[13] Cockayne D. A., Dunn P. M., Zhong Y. (2005). P2X2 Knockout Mice and P2X2/P2X3 Double Knockout Mice Reveal a Role for the P2X2 Receptor Subunit in Mediating Multiple Sensory Effects of ATP. *The Journal of Physiology*.

[14] Montilla A., Mata G. P., Matute C., Domercq M. (2020). Contribution of P2X4 Receptors to CNS Function and Pathophysiology. *International Journal of Molecular Sciences*.

[15] Virgilio F. (2015). P2X Receptors and Inflammation. *Current Medicinal Chemistry*.

[16] Bartlett R., Stokes L., Sluyter R. (2014). The P2X7 Receptor Channel: Recent Developments and the Use of P2X7 Antagonists in Models of Disease. *Pharmacological Reviews*.

[17] Gelin C. F., Bhattacharya A., Letavic M. A. (2020). P2X7 Receptor Antagonists for the Treatment of Systemic Inflammatory Disorders. *Progress in Medicinal Chemistry*.

[18] Marchese P., Lombardi M., Mantione M. E. (2021). Confocal Blood Flow Videomicroscopy of Thrombus Formation Over Human Arteries and Local Targeting of P2X7. *International Journal of Molecular Sciences*.

[19] Cully M. (2020). Can Anti-Inflammatory Strategies Light Up the Dim Depression Pipeline?. *Nature Reviews Drug Discovery*.

[20] Pharmacology IUOBAC (2024). *IUPHAR/BPS Guide to Pharmacology*.

[21] Nedeva C., Menassa J., Puthalakath H. (2019). Sepsis: Inflammation is a Necessary Evil. *Frontiers in Cell and Developmental Biology*.

[22] Giamarellos-Bourboulis E. J., Aschenbrenner A. C., Bauer M. (2024). The Pathophysiology of Sepsis and Precision-Medicine-Based Immunotherapy. *Nature Immunology*.

[23] Yamamoto S., Horita N., Hara J. (2024). Benefit-Risk Profile of P2X3 Receptor Antagonists for Treatment of Chronic Cough: Dose-Response Model-Based Network Meta-Analysis. *Chest*.

[24] Mcgarvey L. P., Birring S. S., Morice A. H. (2022). Efficacy and Safety of Gefapixant, A P2X(3) Receptor Antagonist, in Refractory Chronic Cough and Unexplained Chronic Cough (COUGH-1 and COUGH-2): Results From Two Double-Blind, Randomised, Parallel-Group, Placebo-Controlled, Phase 3 Trials. *The Lancet*.

[25] Obara K., Inaba R., Kawakita M., Murata A., Yoshioka K., Tanaka Y. (2022). Effects of NP-1815-PX, A P2X4 Receptor Antagonist, on Contractions in Guinea Pig Tracheal and Bronchial Smooth Muscles. *Biological and Pharmaceutical Bulletin*.

[26] Kobayashi M., Moro N., Yoshino A. (2023). Inhibition of P2X4 and P2X7 Receptors Improves Histological and Behavioral Outcomes After Experimental Traumatic Brain Injury in Rats. *Experimental and Therapeutic Medicine*.

[27] Zou Z., Wang Q., Zhou M. (2020). Protective Effects of P2X7R Antagonist in Sepsis-Induced Acute Lung Injury in Mice via Regulation of Circ_0001679 and Circ_0001212 and Downstream Pln, Cdh2, and Nprl3 Expression. *The Journal of Gene Medicine*.

[28] Arulkumaran N., Sixma M. L., Pollen S. (2018). P2X(7) Receptor Antagonism Ameliorates Renal Dysfunction in a Rat Model of Sepsis. *Physics Reports*.

[29] Ozkanlar S., Ulas N., Kaynar O., Satici E. (2023). P2X7 Receptor Antagonist A-438079 Alleviates Oxidative Stress of Lung in LPS-Induced Septic Rats. *Purinergic Signalling*.

[30] Zhang R., Su K., Yang L. (2023). Design, Synthesis, and Biological Evaluation of Novel P2X7 Receptor Antagonists for the Treatment of Septic Acute Kidney Injury. *Journal of Medicinal Chemistry*.

[31] Wu X., Ren J., Chen G. (2017). Systemic Blockade of P2X7 Receptor Protects Against Sepsis-Induced Intestinal Barrier Disruption. *Scientific Reports*.

[32] Kim T. S., Choi D. H. (2020). Liver Dysfunction in Sepsis. *Korean Journal of Gastroenterology*.

[33] Woźnica E. A., Inglot M., Woźnica R. K., Łysenko L. (2018). Liver Dysfunction in Sepsis. *Advances in Clinical and Experimental Medicine*.

[34] Roman R. M., Fitz J. G. (1999). Emerging Roles of Purinergic Signaling in Gastrointestinal Epithelial Secretion and Hepatobiliary Function. *Gastroenterology*.

[35] Che M., Gatmaitan Z., Arias I. M. (1997). Ectonucleotidases, Purine Nucleoside Transporter, and Function of the Bile Canalicular Plasma Membrane of the Hepatocyte. *The FASEB Journal*.

[36] Woo K., Sathe M., Kresge C. (2010). Adenosine Triphosphate Release and Purinergic (P2) Receptor-Mediated Secretion in Small and Large Mouse Cholangiocytes. *Hepatology*.

[37] Lecut C., Faccinetto C., Delierneux C. (2012). ATP-Gated P2X1 Ion Channels Protect Against Endotoxemia by Dampening Neutrophil Activation. *Journal of Thrombosis and Haemostasis*.

[38] Graubardt N., Fahrner R., Trochsler M. (2013). Promotion of Liver Regeneration by Natural Killer Cells in a Murine Model is Dependent on Extracellular Adenosine Triphosphate Phosphohydrolysis. *Hepatology*.

[39] Beldi G., Wu Y., Banz Y. (2008). Natural Killer T Cell Dysfunction in CD39-Null Mice Protects Against Concanavalin A-Induced Hepatitis. *Hepatology*.

[40] Ishikawa H., Barber G. N. (2008). STING is an Endoplasmic Reticulum Adaptor That Facilitates Innate Immune Signalling. *Nature*.

[41] Kudira R., Malinka T., Kohler A. (2016). P2X1-Regulated IL-22 Secretion by Innate Lymphoid Cells is Required for Efficient Liver Regeneration. *Hepatology*.

[42] Matos C., Metens T. (2024). Liver Fibrosis: Diving Into Microstructure With Diffusion-Weighted Imaging. *Radiology*.

[43] LE Guilcher C., Garcin I., Dellis O. (2018). The P2X4 Purinergic Receptor Regulates Hepatic Myofibroblast Activation During Liver Fibrogenesis. *Journal of Hepatology*.

[44] Ersahin T., Tuncbag N., Cetin-Atalay R. (2015). The PI3K/AKT/mTOR Interactive Pathway. *Molecular BioSystems*.

[45] Li Z. X., Sheng X. D., Wang Y. L., Wen Lv X. (2022). Blocking P2X4 Purinergic Receptor Attenuates Alcohol-Related Liver Fibrosis by Inhibiting Hepatic Stellate Cell Activation Through PI3K/AKT Signaling Pathway. *International Immunopharmacology*.

[46] Rossato M., Di Vincenzo A., Pagano C., Hadi H. E., Vettor R. (2020). The P2X7 Receptor and NLRP3 Axis in Non-Alcoholic Fatty Liver Disease: A Brief Review. *Cells*.

[47] Fu J., Wu H. (2023). Structural Mechanisms of NLRP3 Inflammasome Assembly and Activation. *Annual Review of Immunology*.

[48] Winkler S., Rösen-Wolff A. (2015). Caspase-1: An Integral Regulator of Innate Immunity. *Seminars in Immunopathology*.

[49] Pelegrin P. (2021). P2X7 Receptor and the NLRP3 Inflammasome: Partners in Crime. *Biochemical Pharmacology*.

[50] Sollberger G., Strittmatter G. E., Garstkiewicz M., Sand J., Beer H. D. (2014). Caspase-1: The Inflammasome and beyond. *Innate Immunity*.

[51] Pang F., Yang Y., Huang S. (2023). Electroacupuncture Alleviates Depressive-Like Behavior by Modulating the Expression of P2X7/NLRP3/IL-1β of Prefrontal Cortex and Liver in Rats Exposed to Chronic Unpredictable Mild Stress. *Brain Sciences*.

[52] Ren W., Rubini P., Tang Y., Engel T., Illes P. (2021). Inherent P2X7 Receptors Regulate Macrophage Functions During Inflammatory Diseases. *International Journal of Molecular Sciences*.

[53] Narahari A. K., Kreutzberger A. J., Gaete P. S. (2021). ATP and Large Signaling Metabolites Flux Through Caspase-Activated Pannexin 1 Channels. *eLife*.

[54] Agnew A., Nulty C., Creagh E. M. (2021). Regulation, Activation and Function of Caspase-11 During Health and Disease. *International Journal of Molecular Sciences*.

[55] de Gassart A., Martinon F. (2015). Pyroptosis: Caspase-11 Unlocks the Gates of Death. *Immunity*.

[56] Li W., Li J., Sama A. E., Wang H. (2013). Carbenoxolone Blocks Endotoxin-Induced Protein Kinase R (PKR) Activation and High Mobility Group Box 1 (HMGB1) Release. *Molecular Medicine*.

[57] Woehrle T., Yip L., Manohar M. (2010). Hypertonic Stress Regulates T Cell Function via Pannexin-1 Hemichannels and P2X Receptors. *Journal of Leukocyte Biology*.

[58] Cayrol C., Girard J. P. (2018). Interleukin-33 (IL-33): A Nuclear Cytokine From the IL-1 Family. *Immunological Reviews*.

[59] Shakerian L., Kolahdooz H., Garousi M. (2022). IL-33/ST2 Axis in Autoimmune Disease. *Cytokine*.

[60] Wang P., Shi B., Wang C. (2022). Hepatic Pannexin-1 Mediates ST2(+) Regulatory T Cells Promoting Resolution of Inflammation in Lipopolysaccharide-Induced Endotoxemia. *Clinical and Translational Medicine*.

[61] Gofton T. E., Young G. B. (2012). Sepsis-Associated Encephalopathy. *Nature Reviews Neurology*.

[62] Hong Y., Chen P., Gao J., Lin Y., Chen L., Shang X. (2023). Sepsis-Associated Encephalopathy: From Pathophysiology to Clinical Management. *International Immunopharmacology*.

[63] Obermeier B., Daneman R., Ransohoff R. M. (2013). Development, Maintenance and Disruption of the Blood-Brain Barrier. *Nature Medicine*.

[64] Kadry H., Noorani B., Cucullo L. (2020). A Blood-Brain Barrier Overview on Structure, Function, Impairment, and Biomarkers of Integrity. *Fluids and Barriers of the CNS*.

[65] Wang Y., Zhu Y., Wang J. (2023). Purinergic Signaling: A Gatekeeper of Blood-Brain Barrier Permeation. *Frontiers in Pharmacology*.

[66] Wang H., Hong L. J., Huang J. Y. (2015). P2RX7 Sensitizes Mac-1/icam-1-Dependent Leukocyte-Endothelial Adhesion and Promotes Neurovascular Injury During Septic Encephalopathy. *Cell Research*.

[67] Rubio-Araiz A., Perez-Hernandez M., Urrutia A. (2014). 3,4-Methylenedioxymethamphetamine (MDMA, Ecstasy) Disrupts Blood-Brain Barrier Integrity Through a Mechanism Involving P2X7 Receptors. *The International Journal of Neuropsychopharmacology*.

[68] Chen S., Tang C., Ding H. (2020). Maf1 Ameliorates Sepsis-Associated Encephalopathy by Suppressing the NF-kB/NLRP3 Inflammasome Signaling Pathway. *Frontiers in Immunology*.

[69] Cowan M., Petri W. A. (2018). Microglia: Immune Regulators of Neurodevelopment. *Frontiers in Immunology*.

[70] Alves V. S., Silva J. P. D., Rodrigues F. C. (2023). P2X7 Receptor Contributes to Long-Term Neuroinflammation and Cognitive Impairment in Sepsis-Surviving Mice. *Frontiers in Pharmacology*.

[71] Santana P. T., Benjamim C. F., Martinez C. G., Kurtenbach E., Takiya C. M., Coutinho-Silva R. (2015). The P2X7 Receptor Contributes to the Development of the Exacerbated Inflammatory Response Associated With Sepsis. *Journal of Innate Immunity*.

[72] Yuan Q., Xie L., Chen C. (2022). Astragalus Polysaccharide Protects Against Blood-Brain Barrier Damage in MCAO Rats by Inhibiting P2X7R Channel. *Nan Fang Yi Ke Da Xue Xue Bao*.

[73] Savio L. E. B., Andrade M. G. J., de Andrade Mello P. (2017). P2X7 Receptor Signaling Contributes to Sepsis-Associated Brain Dysfunction. *Molecular Neurobiology*.

[74] Hirano T. (2021). IL-6 in Inflammation, Autoimmunity and Cancer. *International Immunology*.

[75] Di Virgilio F., Schmalzing G., Markwardt F. (2018). The Elusive P2X7 Macropore. *Trends in Cell Biology*.

[76] Adinolfi E., Callegari M. G., Ferrari D. (2005). Basal Activation of the P2X7 ATP Receptor Elevates Mitochondrial Calcium and Potential, Increases Cellular ATP Levels, and Promotes Serum-Independent Growth. *Molecular Biology of the Cell*.

[77] Burnstock G. (2017). Purinergic Signalling and Neurological Diseases: An Update. *CNS & Neurological Disorders-Drug Targets*.

[78] Huang Z., Weng Y., Shen Q., Zhao Y., Jin Y. (2021). Microplastic: A Potential Threat to Human and Animal Health by Interfering With the Intestinal Barrier Function and Changing the Intestinal Microenvironment. *Science of the Total Environment*.

[79] Peterson L. W., Artis D. (2014). Intestinal Epithelial Cells: Regulators of Barrier Function and Immune Homeostasis. *Nature Reviews Immunology*.

[80] Olivares-Villagómez D., van Kaer L. (2018). Intestinal Intraepithelial Lymphocytes: Sentinels of the Mucosal Barrier. *Trends in Immunology*.

[81] Rodríguez J., Crespo P. (2011). Working Without Kinase Activity: Phosphotransfer-Independent Functions of Extracellular Signal-Regulated Kinases. *Science Signaling*.

[82] Suskiewicz M. J., Prokhorova E., Rack J. G. M., Ahel I. (2023). ADP-Ribosylation From Molecular Mechanisms to Therapeutic Implications. *Cell*.

[83] Hashimoto-Hill S., Friesen L., Kim M., Kim C. H. (2017). Contraction of Intestinal Effector T Cells by Retinoic Acid-Induced Purinergic Receptor P2X7. *Mucosal Immunology*.

[84] Oury C., Wéra O. (2021). P2X1: A Unique Platelet Receptor With a Key Role in Thromboinflammation. *Platelets*.

[85] Oury C., Daenens K., Hu H., Toth‐Zsamboki E., Bryckaert M., Hoylaerts M. F. (2006). ERK2 Activation in Arteriolar and Venular Murine Thrombosis: Platelet Receptor GPIb vs. P2X. *Journal of Thrombosis and Haemostasis*.

[86] He K., Liu X., Hoffman R. D., Shi R. Z., Lv G. Y., Gao J. L. (2022). G-CSF/GM-CSF-Induced Hematopoietic Dysregulation in the Progression of Solid Tumors. *FEBS Open Bio*.

[87] Wéra O., Lecut C., Servais L. (2020). P2X1 Ion Channel Deficiency Causes Massive Bleeding in Inflamed Intestine and Increases Thrombosis. *Journal of Thrombosis and Haemostasis*.

[88] Malmsjö M., Bergdahl A., Möller S. (1999). Congestive Heart Failure Induces Downregulation of P2X1-Receptors in Resistance Arteries. *Cardiovascular Research*.

[89] Tanaka J., Murate M., Wang C. Z., Seino S., Iwanaga T. (1996). Cellular Distribution of the P2X4 ATP Receptor mRNA in the Brain and Non-Neuronal Organs of Rats. *Archives of Histology & Cytology*.

[90] Tani H., Li B., Kusu T. (2021). The ATP-Hydrolyzing Ectoenzyme E-NTPD8 Attenuates Colitis Through Modulation of P2X4 Receptor-Dependent Metabolism in Myeloid Cells. *Proceedings of the National Academy of Sciences of the USA*.

[91] Rong W., Keating C., Sun B., Dong L., Grundy D. (2009). Purinergic Contribution to Small Intestinal Afferent Hypersensitivity in a Murine Model of Postinfectious Bowel Disease. *Neuro-Gastroenterology and Motility*.

[92] Castelucci P., Robbins H. L., Poole D. P., Furness J. B. (2002). The Distribution of Purine P2X(2) Receptors in the Guinea-Pig Enteric Nervous System. *Histochemistry and Cell Biology*.

[93] Ren J., Bian X., Devries M. (2003). P2X2 Subunits Contribute to Fast Synaptic Excitation in Myenteric Neurons of the Mouse Small Intestine. *The Journal of Physiology*.

[94] Schneider R., Leven P., Glowka T. (2021). A Novel P2X2-Dependent Purinergic Mechanism of Enteric Gliosis in Intestinal Inflammation. *EMBO Molecular Medicine*.

[95] Marosti A. R., Da Silva M. V., Palombit K., Mendes C. E., Tavares-de-Lima W., Castelucci P. (2015). Differential Effects of Intestinal Ischemia and Reperfusion in Rat Enteric Neurons and Glial Cells Expressing P2X2 Receptors. *Histology & Histopathology*.

[96] Paulino A. S., Palombit K., Cavriani G. (2011). Effects of Ischemia and Reperfusion on P2X2 Receptor Expressing Neurons of the Rat Ileum Enteric Nervous System. *Digestive Diseases and Sciences*.

[97] Burnstock G. (2014). Purinergic Signalling in the Gastrointestinal Tract and Related Organs in Health and Disease. *Purinergic Signalling*.

[98] Burnstock G. (2016). Purinergic Signalling in the Gut. *Advances in Experimental Medicine and Biology*.

[99] Bian X., Ren J., Devries M. (2003). Peristalsis is Impaired in the Small Intestine of Mice Lacking the P2X3 Subunit. *The Journal of Physiology*.

[100] Schuler A., Wulf D. A., Lu Y. (2018). The Impact of Acute Organ Dysfunction on Long-Term Survival in Sepsis. *Critical Care Medicine*.

[101] Langenberg C., Gobe G., Hood S., May C. N., Bellomo R. (2014). Renal Histopathology During Experimental Septic Acute Kidney Injury and Recovery. *Critical Care Medicine*.

[102] Maiden M. J., Otto S., Brealey J. K. (2016). Structure and Function of the Kidney in Septic Shock. A Prospective Controlled Experimental Study. *American Journal of Respiratory and Critical Care Medicine*.

[103] Carlström M., Wilcox C. S., Arendshorst W. J. (2015). Renal Autoregulation in Health and Disease. *Physiological Reviews*.

[104] Nishiyama A., Majid D. S., Taher K. A., Miyatake A., Navar L. G. (2000). Relation Between Renal Interstitial ATP Concentrations and Autoregulation-Mediated Changes in Renal Vascular Resistance. *Circulation Research*.

[105] Franco M., Pérez-Méndez O., Kulthinee S., Navar L. G. (2019). Integration of Purinergic and Angiotensin II Receptor Function in Renal Vascular Responses and Renal Injury in Angiotensin II-Dependent Hypertension. *Purinergic Signalling*.

[106] Menzies R. I., Tam F. W., Unwin R. J., Bailey M. A. (2017). Purinergic Signaling in Kidney Disease. *Kidney International*.

[107] Chan C. M., Unwin R. J., Bardini M. (1998). Localization of P2X1 Purinoceptors by Autoradiography and Immunohistochemistry in Rat Kidneys. *American Journal of Physiology-Renal Physiology*.

[108] Osmond D. A., Inscho E. W. (2010). P2X(1) Receptor Blockade Inhibits Whole Kidney Autoregulation of Renal Blood Flow In Vivo. *American Journal of Physiology-Renal Physiology*.

[109] Guan Z., Giddens M. I., Osmond D. A. (2013). Immunosuppression Preserves Renal Autoregulatory Function and Microvascular P2X(1) Receptor Reactivity in ANG II-Hypertensive Rats. *American Journal of Physiology-Renal Physiology*.

[110] Suurväli J., Boudinot P., Kanellopoulos J., Rüütel Boudinot S. (2017). P2X4: A Fast and Sensitive Purinergic Receptor. *Biomedical Journal*.

[111] Wildman S. S., Marks J., Turner C. M. (2008). Sodium-Dependent Regulation of Renal Amiloride-Sensitive Currents by Apical P2 Receptors. *Journal of the American Society of Nephrology*.

[112] Craigie E., Menzies R. I., Larsen C. K. (2018). The Renal and Blood Pressure Response to Low Sodium Diet in P2X4 Receptor Knockout Mice. *Physics Reports*.

[113] de Baaij J. H., Kompatscher A., Viering D. H., Bos C., Bindels R. J., Hoenderop J. G. (2016). P2X6 Knockout Mice Exhibit Normal Electrolyte Homeostasis. *PLoS One*.

[114] Qian Y., Zhao N., Wang M., Zou Z., Xie K. (2024). P2X7 Receptor Deficiency Attenuates Cisplatin-Induced Kidney Injury via Inhibiting NLRP3 Inflammasome Activation. *Biochemical Pharmacology*.

[115] Solini A., Menini S., Rossi C. (2013). The Purinergic 2X7 Receptor Participates in Renal Inflammation and Injury Induced by High-Fat Diet: Possible Role of NLRP3 Inflammasome Activation. *The Journal of Pathology*.

[116] Siamantouras E., Price G. W., Potter J. A., Hills C. E., Squires P. E. (2019). Purinergic Receptor (P2X7) Activation Reduces Cell-Cell Adhesion Between Tubular Epithelial Cells of the Proximal Kidney. *Nanomedicine: Nanotechnology, Biology and Medicine*.

[117] Nespoux J., Monaghan M. L. T., Jones N. K. (2024). P2X7 Receptor Knockout Does Not Alter Renal Function or Prevent Angiotensin II-Induced Kidney Injury in F344 Rats. *Scientific Reports*.

[118] Sessler C. N., Bloomfield G. L., Fowler A. A. (1996). Current Concepts of Sepsis and Acute Lung Injury. *Clinics in Chest Medicine*.

[119] Bos L. D. J., Ware L. B. (2022). Acute Respiratory Distress Syndrome: Causes, Pathophysiology, and Phenotypes. *The Lancet*.

[120] Myrtek D., Müller T., Geyer V. (2008). Activation of Human Alveolar Macrophages via P2 Receptors: Coupling to Intracellular Ca2+ Increases and Cytokine Secretion. *Journal of Immunology*.

[121] Alveal M., Méndez A., García A., Henríquez M. (2024). *Purinergic Regulation of Pulmonary Vascular Tone*.

[122] Gui Y., Wang Z., Sun X. (2011). Uridine Adenosine Tetraphosphate Induces Contraction of Airway Smooth Muscle. *American Journal of Physiology-Lung Cellular and Molecular Physiology*.

[123] Visovatti S. H., Hyman M. C., Goonewardena S. N. (2016). Purinergic Dysregulation in Pulmonary Hypertension. *American Journal of Physiology-Heart and Circulatory Physiology*.

[124] Théâtre E., Frederix K., Guilmain W. (2012). Overexpression of CD39 in Mouse Airways Promotes Bacteria-Induced Inflammation. *The Journal of Immunology*.

[125] Rich P. B., Douillet C. D., Mahler S. A., Husain S. A., Boucher R. C. (2003). Adenosine Triphosphate is Released During Injurious Mechanical Ventilation and Contributes to Lung Edema. *The Journal of Trauma, Injury, Infection, and Critical Care*.

[126] Adriaensen D., Timmermans J. P. (2004). Purinergic Signalling in the Lung: Important in Asthma and COPD?. *Current Opinion in Pharmacology*.

[127] Pelleg A., Schulman E. S., Barnes P. J. (2018). Adenosine 5′-Triphosphate’s Role in Bradycardia and Syncope Associated With Pulmonary Embolism. *Respiratory Research*.

[128] Pelleg A., Xu F., Zhuang J., Undem B., Burnstock G. (2019). DT-0111: A Novel Drug-Candidate for the Treatment of COPD and Chronic Cough. *Therapeutic Advances in Respiratory Disease*.

[129] Basoglu O. K., Barnes P. J., Kharitonov S. A., Pelleg A. (2015). Effects of Aerosolized Adenosine 5′-Triphosphate in Smokers and Patients With COPD. *Chest*.

[130] Bonvini S. J., Birrell M. A., Grace M. S. (2016). Transient Receptor Potential Cation Channel, Subfamily V, Member 4 and Airway Sensory Afferent Activation: Role of Adenosine Triphosphate. *Journal of Allergy and Clinical Immunology*.

[131] Adriaensen D., Brouns I., Timmermans J. P. (2015). Sensory Input to the Central Nervous System From the Lungs and Airways: A Prominent Role for Purinergic Signalling via P2X2/3 Receptors. *Autonomic Neuroscience*.

[132] Hadley S. H., Bahia P. K., Taylor-Clark T. E. (2014). Sensory Nerve Terminal Mitochondrial Dysfunction Induces Hyperexcitability in Airway Nociceptors via Protein Kinase C. *Molecular Pharmacology*.

[133] Fois G., Winkelmann V. E., Bareis L. (2018). ATP is Stored in Lamellar Bodies to Activate Vesicular P2X(4) in an Autocrine Fashion Upon Exocytosis. *Journal of General Physiology*.

[134] Thompson K. E., Korbmacher J. P., Hecht E. (2013). Fusion-Activated Cation Entry (FACE) via P2X_4_ Couples Surfactant Secretion and Alveolar Fluid Transport. *The FASEB Journal*.

[135] Hasan D., Satalin J., van der Zee P. (2018). Excessive Extracellular ATP Desensitizes P2Y2 and P2X4 ATP Receptors Provoking Surfactant Impairment Ending in Ventilation-Induced Lung Injury. *International Journal of Molecular Sciences*.

[136] Lucattelli M., Cicko S., Müller T. (2011). P2X7 Receptor Signaling in the Pathogenesis of Smoke-Induced Lung Inflammation and Emphysema. *American Journal of Respiratory Cell and Molecular Biology*.

[137] Osuchowski M. F., Ayala A., Bahrami S. (2018). Minimum Quality Threshold in Pre-Clinical Sepsis Studies (MQTiPSS): An International Expert Consensus Initiative for Improvement of Animal Modeling in Sepsis. *Intensive Care Medicine Experimental*.

